# A Systematic Review of Pharmacovigilance Systems in Developing Countries Using the WHO Pharmacovigilance Indicators

**DOI:** 10.1007/s43441-022-00415-y

**Published:** 2022-06-03

**Authors:** Hamza Y. Garashi, Douglas T. Steinke, Ellen I. Schafheutle

**Affiliations:** Division of Pharmacy and Optometry, School of Health Sciences, Faculty of Biology, Medicine and Health, The University of Manchester, Manchester, M13 9PT UK

**Keywords:** Pharmacovigilance, Developing countries, Evaluation studies, Programme evaluation, Benchmarking

## Abstract

**Background:**

In the context of the growth of pharmacovigilance (PV) among developing countries, this systematic review aims to synthesise current research evaluating developing countries’ PV systems’ performance.

**Methods:**

EMBASE, MEDLINE, CINAHL Plus and Web of Science were searched for peer-reviewed studies published in English between 2012 and 2021. Reference lists of included studies were screened. Included studies were quality assessed using Hawker et al.'s nine-item checklist; data were extracted using the WHO PV indicators checklist. Scores were assigned to each group of indicators and used to compare countries’ PV performance.

**Results:**

Twenty-one unique studies from 51 countries were included. Of a total possible quality score of 36, most studies were rated medium (*n* = 7 studies) or high (*n* = 14 studies). Studies obtained an average score of 17.2 out of a possible 63 of the WHO PV indicators. PV system performance in all 51 countries was low (14.86/63; range: 0–26). Higher average scores were obtained in the ‘Core’ (9.27/27) compared to ‘Complementary’ (5.59/36) indicators. Overall performance for ‘Process’ and ‘Outcome’ indicators was lower than that of ‘Structural’.

**Conclusion:**

This first systematic review of studies evaluating PV performance in developing countries provides an in-depth understanding of factors affecting PV system performance.

**Supplementary Information:**

The online version contains supplementary material available at 10.1007/s43441-022-00415-y.

## Introduction

Pharmacovigilance (PV) with its ultimate goal of minimising risks and maximising the benefits of medicinal products serves as an important public health tool [1, 2]. The World Health Organization (WHO) defines PV as “the science and activities relating to the detection, assessment, understanding and prevention of adverse effects or any other drug-related problem”(p. 7) [3].

Prior to approval by regulatory authorities, drug products are required to undergo extensive testing and rigorous evaluation during clinical trials, to establish their safety and efficacy [4, 5]. The rationale for post-marketing PV is based on the need to mitigate the limitations of pre-marketing/registration clinical trials including small population sizes, a short length of time and the exclusion of special population groups (e.g. pregnant women and children) [6, 7]. Therefore, unexpected or severe adverse drug reactions (ADRs) are often not identified before regulatory approval resulting in increased morbidity, mortality and financial loss [8, 9]. PV allows for the post-marketing (i.e. real-world) collection of drug safety and efficacy information thereby reducing patients' drug-related morbidity and mortality [10]. Moreover, PV reduces the financial costs associated with the provision of care for patients affected by such problems [11, 12]. This is achieved by communicating medicines’ risks and benefits thus enhancing medication safety at various levels of the healthcare system [13] as well as providing information and knowledge informing regulatory actions [14–16]. It is important to note that PV activities are not limited to protecting patient safety in the post-marketing phase but apply to a drug product’s entire lifecycle and are a continuation and completion of the analysis performed on medicines from the pre-registration clinical trials [17]. PV also plays a role in helping drug manufacturing firms in carrying out patient outreach through communicating with patients about drug products’ risk–benefit profile thus making them better informed and building their trust in the industry [18]. As the collective payers for drug products, insurance firms rely on PV information as a measure of drug products’ demonstrated value to patients in making decisions about reimbursement [18, 19].

PV systems’ differences in developing countries are influenced by local contextual factors such as healthcare expenditure, disease types and prevalence, and political climate [20]. These differences can lead to variability in medicine use and the profile of adverse effects suffered by patients which makes it essential that every country establish its own PV system [21]. Most developed countries started PV activities after the thalidomide disaster in the 1960s by establishing PV systems and joining the WHO Programme for International Drug Monitoring (PIDM) [22–24]. Developing countries did not join the PIDM until the 1990s or later [22–24], but since then, the number of developing countries implementing PV and joining WHO PIDM has steadily increased [23, 24].

Over the past few decades, both national and international legislative organisations, as well as national medicines regulatory authorities (NMRAs) have published a considerable amount of legislation and guidance to provide countries with a legal foundation and practical implementation guidance for national PV systems [25]. Among these is the Guidelines on Good Pharmacovigilance Practices (GVP) implemented by the European medicines agency (EMA) in 2012 which aim to facilitate the performance of PV in the European Union (EU) [26]. Many developing countries wishing to align their new and evolving national PV frameworks with international standards use the EMA’s GVP guidelines as a reference for setting up their national PV systems [25, 27].

The WHO recommends that PV systems incorporate evaluation and assessment mechanisms with specific performance criteria [28]. Despite the growth in PV development and practice among developing countries, a gap remains in efforts to assess, evaluate, and monitor their systems’ and activities’ status, growth, and impact [29]. To promote patient safety and enhance efforts aimed at strengthening PV systems in developing countries with nascent PV systems, it is imperative to assess existing conditions [13, 30]. Such assessment can help define the elements of a sustainable PV strategy and areas for improvements as the basis to plan for improved public health and safety of medicines [13, 29, 31].

This review aims to systematically identify published peer-reviewed research that evaluates the characteristics, performance, and/or effectiveness of PV systems in developing countries.

## Methods

This systematic review was conducted in accordance with the Preferred Reporting Items for Systematic Reviews and Meta-analyses (PRISMA) statement [32]. A PRISMA checklist is included in Online Resource 1.

### Theoretical Framework

As a theoretical framework, this study adopted the WHO PV indicators, which measure inputs, processes, outputs, outcomes, and impacts. These WHO indicators “provide information on how well a pharmacovigilance programme is achieving its objectives” (p. 4) [30]. Details on how the WHO PV indicators were derived and validated have been described by Isah and Edwards [29]. The indicator-based pharmacovigilance assessment tool (IPAT) was considered but not chosen because its sensitivity and specificity as a measurement tool have not been established [33].

There are 63 WHO PV indicators, which are classified into three main types: 1—Structural (21 indicators): assess the existence of key PV structures, systems and mechanisms; 2—Process (22 indicators): assess the extent of PV activities, i.e. how the system is operating; 3—Outcome/impact (20 indicators): measure effects (results and changes), i.e. the extent of realisation of PV objectives [30]. Each of these types is further subdivided into two categories: 1—Core (total 27) indicators are considered highly relevant, important and useful in characterising PV, and 2—Complementary (total 36) are additional measurements that are considered relevant and useful [30].

### Information Sources and Search Strategy

Four electronic databases (EMBASE, MEDLINE, CINAHL Plus and Web of Science) were searched for international peer-reviewed research evidence published between 1st January 2012 (the year when the EMA’s guidelines on GVP were due for implementation) and 16th July 2021. The search was initiated using the term ‘pharmacovigilance’ and its synonyms in combination with other groups of keywords that covered ‘evaluation’. The search terms are listed in Table 1 (see Online Resource 2 for search strategy). Reference lists of included studies were also screened.

**Table 1:** Keywords used for the search

Keyword	Search terms
Pharmacovigilance	Pharmacovigilance OR Drug Surveillance Program OR drug safety OR adverse drug reactions reporting systems OR post-marketing surveillance
Evaluation	Evaluat* OR Monitor* OR Assess* OR Benchmark*

### Data Screening

Once all duplicate titles had been removed, screening of abstracts and then full texts against the inclusion/exclusion criteria (Table 2) was conducted by the lead author. Both co-authors were consulted where queries arose, and the decision on which articles to include in the review was discussed and agreed upon by all authors.

**Table 2:** Inclusion and exclusion criteria

	Inclusion criteria	Exclusion criteria
Setting	Developing countries	
Species	Human	Animal
Location	International	
Language	English	
Design/Study type	Qualitative and quantitative studies. Randomised control trials (RCTs) with a primary component related to the evaluation or assessment of pharmacovigilance systems or activities	All types of reviews. Randomised control trials (RCTs) with no secondary aim related to the evaluation of pharmacovigilance systems or activities
Publication type	Full-text peer-reviewed journal studies based on empirical research or with a clear empirical base	Non-peer-reviewed studies and conference abstracts, case reports, editorials, opinion pieces, commentaries and conceptual studies
Publication date	2012–2021	
Focus of study	Studies about the characteristics, performance metrics, or effectiveness of pharmacovigilance system(s) at some level. e.g. PV centre (national or peripheral), healthcare facilities (hospitals or clinics), Public Healthcare Programmes (PHP), or pharmaceutical companies within a developing country	∙ Studies focussing on non-medication related adverse events (e.g. surgical adverse events), allergies, medication errors, abuse or misuse, medical devices, veterinary products, traditional or complementary medicines, vaccines, food supplements
		∙ ADR-reporting systems based on computerised physician order entry systems, electronic medical records and registries specific to one drug or disease
		∙ Studies of pharmacodynamic, pharmacokinetic and pharmacogenetic measures

### Data Extraction, Synthesis and Quality Assessment

Data were extracted independently by the lead author and checked by the co‐authors, using a data extraction tool based on the WHO PV indicators checklist. Data were extracted at two levels: overall study and studied country/countries. For each study, data were extracted related to which of the WHO PV indicators the study provided information, while for individual countries assessed in the studies, data (qualitative and quantitative) relating to each indicator were extracted. The data were placed into Microsoft Excel and NVivo and analysed thematically to aid comparison between studies and particular countries.

A scoring system was developed for the purpose of this review to quantify the indices thus highlighting countries’ PV system strengths and deficiencies in numerical terms. Each of the 63 indicators was scored separately and a final score was calculated for each study. If information relating to an indicator was present, a score of 1 was given. A score of 0 was given where data were not provided, missing, not applicable or not clear. Where information for a particular country was provided by more than one study, the latest study was used. In cases where country data were available for more than one system level (e.g. national level and institutional level), the information from the higher level was used. The final scores were used to benchmark national PV performance and compare countries both within and across regions.

The quality of included studies was evaluated using Hawker et al.’s nine‐item checklist [34] for appraising disparate studies. The checklist allows scoring of individual parameters and a total score that allows the comparison of strengths and weaknesses within and across studies. Total scores could range from 9 to 36, by scoring studies as “Good” (4), “Fair” (3), “Poor” (2), “Very poor” (1) for each checklist item (title, introduction and aims, method and data, sampling, data analysis, ethics and bias, results, transferability or generalisability, implications and usefulness). To categorise the sum quality ranking of studies, previously used cut-offs were adopted: [35, 36] high (30–36 points), medium (24–29 points) and low quality (9–23 points).

## Results

**Fig. 1:**
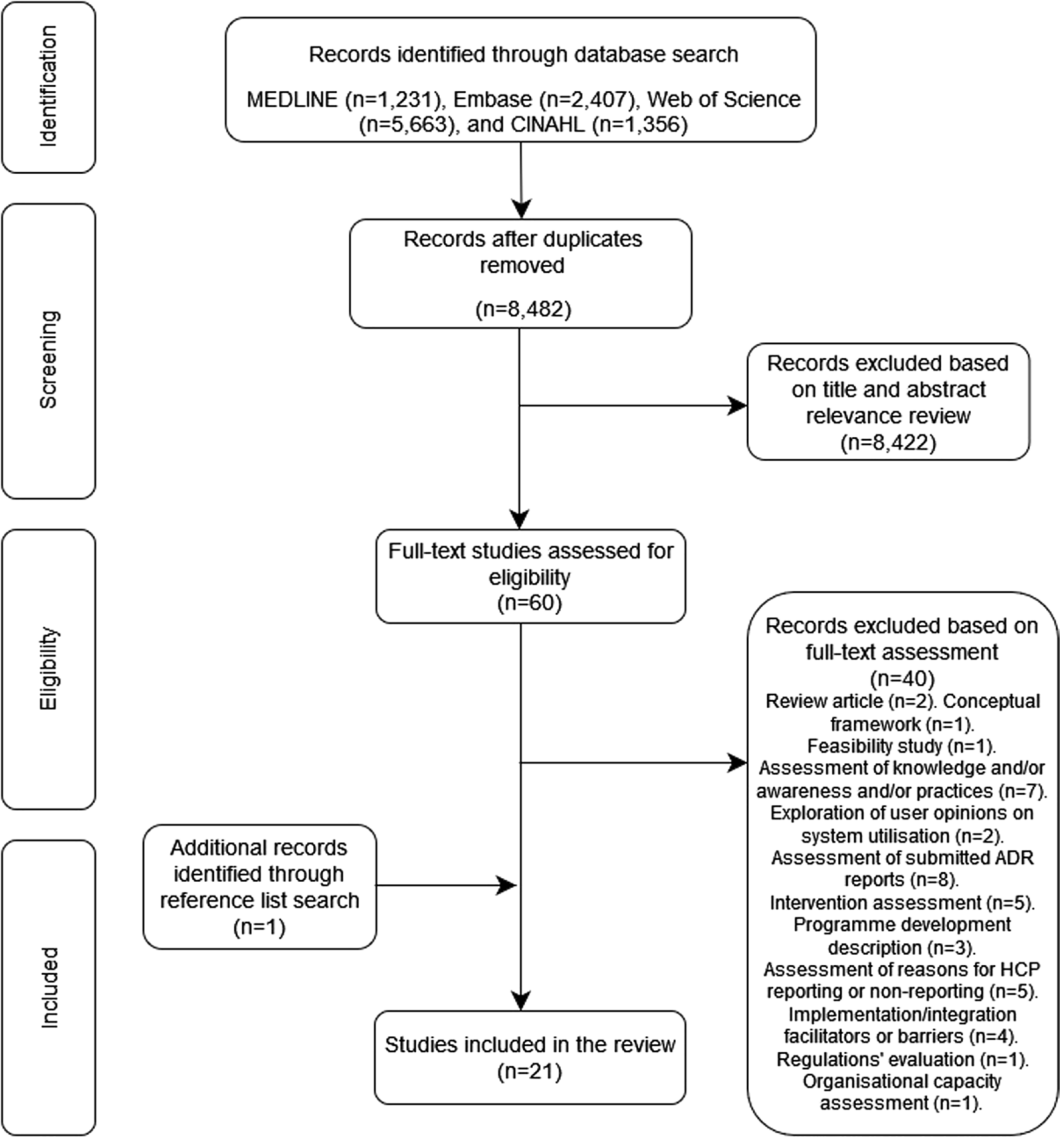
Flow diagram of studies included/excluded in the systematic review

Following the removal of duplicates (*n* = 2175), 8482 studies were screened, with 8462 studies excluded following title, abstract, and full-text review. Screening of reference lists of the remaining studies (*n* = 20) lead to a total of 21 included studies. Figure 1 presents a PRISMA flowchart demonstrating this process.

### Study Characteristics

The 21 included studies (Table 3) evaluated PV systems in 51 countries across single or multiple countries’ National PV Centres (NPVCs), Public Health Programmes (PHPs), healthcare facilities (e.g. hospitals) or pharmaceutical companies. Most of the studies (*n* = 13) had been published since 2016. Eleven studies focusesd on African countries [37–47] with one of these also including India [42]. Four studies involved Middle Eastern and/or Eastern Mediterranean countries [48–51], and four covered East or South-East Asian countries [52–55]. One study dealt with countries in the Asia–Pacific region [56] and one study focussed on a country in South America [57].

**Table 3:** Summary of details of included studies and quality assessment scores

Author(s) and publication year	Study aim	Study design	Study setting	Pharmacovigilance system level	Sample size	Methods	Evaluation tool(s)	Aspects evaluated by the study	Study limitations	Quality Score (out of 36)
Abiri and Johnson [37]	To evaluate the current status of PV in Sierra Leone through a comprehensive and system-based approach that covered the Pharmacy Board of Sierra Leone, healthcare facilities and Public Health Programmes	Descriptive cross-sectional study	Sierra Leone	National medicines regulatory authority, health facilities, and Public Health Programmes (PHPs)	14 Participants	Structured interviews with key informants from the Pharmacy Board of Sierra Leone (PBSL), six hospitals, and six Public Health Programmes (PHPs), as well documentary review	Indicator-Based Pharmacovigilance Assessment Tool (IPAT)	1—Policy, law and regulation; 2—Systems, structures and stakeholder coordination; 3—Signal generation and data management; 4—Risk assessment and evaluation; and 5—Risk management and communication	Small sample size recruited through convenience sampling. Use of a score of 60% as a threshold for the overall functionality of the pharmacovigilance system despite no evidence from IPAT	30
Allabi and Nwokike [38]	To draw up a portrait of policy documents and practical actions in the areas of PV, quality control of Artemisinin-based Combination Therapies (ACTs) and monitoring of resistance of ACT in Republic of Benin (situational analysis), identification of the main barriers which prevent their implementation and the discussion focus on the recommendations for towards the establishment of an effective and functional PV system in Benin	Not reported	Republic of Benin	PV systems in drug regulation system (DPM), National malaria control programme (NMCP), known as “Programme National de Lutte Contre le Paludisme” (PNLP) in Benin), quality control of drugs centre (LNCQ) and the biggest teaching hospital (CNHU)	68 physicians, 45 pharmacists and 43 pharmaceutical company representatives, key informants from the National Laboratory of Drugs Control Quality (LNCQ), Directorate of Pharmacy and Drug Regulations (DPM), National Malaria Control Programme (NMCP) and the Director of the teaching hospital in Cotonou: Centre National Hospitalier Universitaire (CNHU)	Interviewer administered semi-structured questionnaire with physicians, pharmacists, and pharmaceutical company representatives; focus groups and structured interviews with representatives from the NMCP (Programme National de Lutte Contre le Paludisme (PNLP)), the National Laboratory of Drugs Control Quality (Laboratoire National de Control de Qualité (LNCQ)), DPM and the director of the CNHU-teaching hospital; and documentary review	Semi-structured questionnaire based on adverse drug reaction reporting and reasons for non-reporting; no framework reported for focus groups; structured interviews and documentary review based on Indicator-Based Pharmacovigilance Assessment Tool (IPAT); SWOT analysis	Semi-structured questionnaire: knowledge, attitude and practice relating to spontaneous reporting of adverse drug reactions, specific questions examining the ADRs related to Artemisinin-based Combination Therapy (ACT), reasons for non-reporting and important factors in a decision to report; focus groups: Assess the practice and problems in the pharmacovigilance system and quality control of ACTs and ways to solve these problems; structured interviews and document review: 1—Policy, law and regulation; 2—Systems, structures and stakeholder coordination; 3—Signal generation and data management; 4—Risk assessment and evaluation; and 5—Risk management and communication; strengths, weaknesses, opportunities and threats used to make recommendations	Not reported	28
Alshammari et al. [48]	To investigate and provide an overview of the current situation and on the activities of the national pharmacovigilance centres in Arab countries	Cross-sectional study	Arab countries (members of the League of Arab States)	National Pharmacovigilance Centres	15 countries: Algeria (AL), Egypt (EG), Jordan (JO), Iraq (IQ), Kuwait (KW), Libya (LB), Lebanon (LE), Morocco (MA), Oman (OM), Palestine (PA), Kingdom of Saudi Arabia (KSA), Sudan (SU), Tunisia (TN), United Arab Emirates (UAE), and Yemen (YE)	Self-administered questionnaire by representatives of National Pharmacovigilance Centres	A previously conducted survey carried out by WHO Uppsala Monitoring Centre (UMC)	1—Country and respondent background information; 2—Overview of the PV programme; 3—Spontaneous reporting; 4—PV activities; 5—level of support, including funding, staff, and software; 6—Usefulness of information from PV activities; and 7—Registry availability; also, presence of a designated national centre/department that conducts PV activities	Pertinent information missing. Programme features and development plans might have changed since the time of the study. Not all countries responded	31
Barry et al. [39]	To conduct a comparative assessment of the current national PV system at the respective National Medicines Regulatory Authorities in Ethiopia, Kenya, Rwanda, and Tanzania for future targeted capacity-building interventions to be carried out by the PROFORMA project	Cross-sectional descriptive study	Ethiopia (ET), Kenya (KE), Rwanda (RW), and Tanzania (TZ)	National Pharmacovigilance Centres housed within the National Medicines Regulatory Authorities	Between two and four NMRA staff members working in PV from each country	Structured interviews with key informants (NMRA staff working in PV) and documentary review	East African Community (EAC) Harmonized Pharmacovigilance Indicators tool (derived from the WHO pharmacovigilance indicators and the IPAT) supplemented with a few additional indicators from the WHO Global Benchmarking Tool (GBT) for evaluation of national regulatory systems	EAC Indicators tool: 1—Policy, law, and regulation; 2—Systems, structures, and stakeholder coordination; 3—Signal generation and data management; 4—Risk assessment and evaluation; and 5—Risk management and communication; WHO Global Benchmarking Tool: 1—Guidelines ensuring encouragement of different stakeholders to report ADRs and AEs to the Marketing Authorisation Holder (MAH) and/or NMRA; 2—Legal provisions and regulations allowing NMRA to require safety and effectiveness studies; 3—Legal provisions, regulations, and guidelines requiring designation of a person as in charge of the vigilance system	Findings for some of the indicators may have changed since the assessment. Some personal knowledge, experience, and opinions of the regulators were not possible to verify from other sources	30
Barry et al. [40]	To assess and compare the pharmacovigilance systems and practices within the Neglected Tropical Disease (NTD) programmes in Ethiopia, Kenya, Rwanda, and Tanzania	Cross-sectional descriptive study	Ethiopia (ET), Kenya (KE), Rwanda (RW), and Tanzania (TZ)	Public Health Programmes	2–3 National NTD programme staff members in Kenya, Tanzania, and Rwanda, and 1 from Ethiopia	Structured interviews with key informants (staff members from the national NTD programme) and documentary review	East African Community (EAC) Harmonized Pharmacovigilance Indicators tool for Public Health Programmes (PHPs) (derived from the WHO pharmacovigilance indicators and the IPAT)	1—Systems, structures, and stakeholder coordination; 2—Signal generation and data management; 3—Risk assessment and evaluation; and 4—Risk management and communication	Not possible to verify all of the information gathered through structured interviews	30
Chan et al. [52]	To review the status of the development of pharmacovigilance in the Association of Southeast Asian Nations (ASEAN) and the relevance of quantitative signal detection algorithms (QSDA) in the ASEAN context. Also to compare the findings in these countries against the more established agencies in Australia, Canada, Japan, South Korea, Switzerland, the UK and the US	Not reported	ASEAN member countries and a group of non-ASEAN countries having close working relations in the area of PV with Singapore: Australia, Canada, Japan, South Korea, Switzerland, UK, and the USA	National Pharmacovigilance Centre	16 countries: 9 ASEAN countries with Myanmar excluded: Brunei Darussalam (BR), Cambodia (KH), Indonesia (ID), Lao People’s Democratic Republic (LA), Malaysia (MY), Philippines (PH), Singapore (SG), Thailand (TH), and Vietnam (VT); and 7 non-ASEAN countries: Australia (AU), Canada (CA), Japan (JP), South Korea (SK), Switzerland (CH), UK, and the USA	Self-administered questionnaire by representatives of National Pharmacovigilance Centres	No tool specified for the questionnaire	1—An overview of the national PV programme; 2—Range of PV activities; 3—Spontaneous ADR reporting and size of the ADR records; 4—Source of ADR information—the importance of the different post-marketing surveillance tools for safety monitoring; 5—Management of ADR reports and signal detection; and 6—The relevance of a QSDA in their respective countries	Limited the survey to all ASEAN countries and seven non-ASEAN countries. A more comprehensive comparison would be to survey a representative sample from all other countries to make a comparison of the status of PV in the ASEAN. Survey responses were focussed on QSDAs and tools only. There was no testing of the reliability of the questionnaire. A substantial number of the survey questions were descriptive. The study did not capture the types and volume of medicines used in the various countries	31
Ejekam et al. [47]	Assess the structures, processes, and outcomes of pharmacovigilance activities in three selected public health programmes (National Malaria, Tuberculosis (TB), HIV/AIDS) in Nigeria using the WHO Pharmacovigilance Indicators and identify possible challenges to achieving the outcomes	Cross-sectional mixed-method study	Nigeria	Public Health Programmes	National PV centre and 3 Public Health Programmes	Structured and semi-structured interviews with key informants from National PV Centre and PHPs and documentary review	WHO Pharmacovigilance Indicators	1—PV structures, processes, and outcomes of each of the PHPs, 2—Efforts and challenges towards achieving the desired PV outcomes from the key informants’ perspectives	Poor recording keeping undermining comprehensive documentation	30
Kabore et al. [41]	To evaluate Burkina Faso’s early-stage drug safety monitoring system through a comprehensive system-based approach	Descriptive cross-sectional study	Burkina Faso	National Medicines Regulatory Authority (NMRA), public health programmes (PHPs) and hospitals	16 participants (1–3 participants per institution)	Structured interviews with key informants from the National Medicines Regulatory Authority (NMRA), six PHPs, and five hospitals, as well as documentary review	Indicator-Based Pharmacovigilance Assessment Tool (IPAT)	1—Policy, law and regulation; 2—Systems, structures and stakeholder coordination; 3—Signal generation and data management; 4—Risk assessment and evaluation; and 5—Risk management and communication; and opinions regarding the current PV system	IPAT limitations: 1. IPAT’s sensitivity and specificity have not been established; 2. Possible imprecision in the quantification of responses in the scoring process; 3. The assessment was reliant on respondents’ declarations; 4. Local adaptation may be necessary due to the tool’s limited testing and validation. Limitations related to evaluation process: Generalisability and reproducibility of the study may be affected due to limited sample in number and diversity	33
Kaewpanukrungsi and Anantachoti [54]	To assess the performance of the Thai National Pharmacovigilance Centre (NPVC) to identify gaps and areas for future improvement	Not reported	Thailand	National Pharmacovigilance Centre	10 participants (8 from the national pharmacovigilance centre and 2 executive staff from the Thai FDA)	Interviews (using semi-structured questionnaires) with and observation of NPVC staff, in-depth interviews with Thai FDA executive staff, and documentary analysis	Open-ended questions: Domains and indicators for NPVC performance assessment	1—Policy, law, plan and structural support, 2—Safety surveillance, 3—Risk management, and 4—Communication of safety information	Not reported	26
Maigetter et al. [42]	To describe the PV systems in India, Uganda, and South Africa. Also, to analyse the extent to which the three countries conformed to the minimum pharmacovigilance requirements by the WHO	Not reported	India (IN), Uganda (UG), and South Africa (SA)	National Pharmacovigilance Centres in Uganda and South Africa, and Regional Pharmacovigilance Centres in Maharashtra State, India	39 participants (20 from India, 8 from Uganda, and 11 from South Africa)	Documentary review of academic literature and policy reports, and interviews with key informants	WHO minimum requirements for functional pharmacovigilance system	Documentary review: pharmaceutical regulation, including regulatory frameworks and capacity; use of medicines; and PV, including descriptions of the adverse event (AE) reporting systems. Interviews: Regulatory systems and policies concerning PV	Reliance on interviews with key informants. Some details regarding budget and staff, as well as composition and functioning of the national advisory committee, were not uniformly available	33
Mugauri et al. [44]	To evaluate the antiretroviral- adverse drug reaction (ARV-ADR) surveillance system in Harare City to identify the reasons for underreporting and recommend solutions	Descriptive cross-sectional study and surveillance system evaluation	Harare City, Zimbabwe	National Pharmacovigilance Centre	52 Health Personnel involved in the ARV-ADR surveillance from 2 hospitals and 17 clinics	Documentary review of patient records and notification forms issued by the hospitals and clinics, as well as interviews with healthcare workers using an interviewer-administered questionnaire	Updated Centres for Disease Control and Prevention (CDC) guidelines for Evaluating Public Health Surveillance Systems and checklist derived from the WHO assessment criteria for a PV system’s stability status (WHO PV Indicators)	Questionnaire: determine health workers' knowledge of the operations and usefulness of the surveillance system; Checklist: evaluates the availability of reporting forms, case definitions and means for communication. Patient records: number of ARV-ADR cases documented, captured, and missed by the surveillance system. Hospital and clinic notifications: evaluating system simplicity, data quality, completeness, acceptability, sensitivity, timeliness and representativeness. PV indicator checklist: core as well as complimentary process indicators, and core outcome indicators	Not reported	29
Muringazuva et al. [43]	To evaluate the Adverse Drug Reaction Surveillance System (ADRSS) to assess the system performance and reasons for not notifying on time	Descriptive cross-sectional study and surveillance system evaluation	Kadoma City, Zimbabwe	Regional Pharmacovigilance System	47 health workers from six health facilities which offered Mass Drug Administration (MDA)	Interviewer administered questionnaire, checklists, and record review (outpatient registers, reports on the ADRSS, meetings' minutes)	Updated Centres for Disease Control Prevention (CDC) Guidelines for Evaluating Public Health Surveillance Systems	System simplicity, stability, acceptability, and completeness; Interviewer administered questionnaire information on health worker knowledge on the ADRSS and to assess the attributes of the ADRSS; checklist was used to assess for the availability of the resources needed for running the ADRSS	Availability of only one notification made it difficult to assess the quality of data	34
Mustafa et al. [51]	To investigate the adverse drug reaction (ADR) reporting system and to suggest possible ways of improving the method of reporting	Prospective observational study	Lahore, Pakistan	Regional health facilities (hospitals)	84 Doctors and 52 Pharmacists from 30 different hospitals in Lahore	Structured interviews using investigator administered questionnaires	A questionnaire based on different ADR systems of developed countries, literature evaluation, and published research articles	Questionnaire 1: General hospital information including ADR systems; Questionnaire 2: Doctors’ and pharmacists’ demographics, knowledge, and attitude to ADR reporting	Not reported	25
Nwaiwu et al. [45]	To evaluate pharmacovigilance practices in pharmaceutical companies in Nigeria	Descriptive study	Lagos, Nigeria	Pharmaceutical Companies	31 companies	Self-administered questionnaire distributed to designated company staff	Questionnaire adapted from existing drug safety laws and guidance and online pharmacovigilance auditing checklists	Basic pharmacovigilance requirements	The sampling method used is prone to selection bias and sampling error. The companies that participated in the study may have differed from companies that did not	27
Opadeyi et al. [46]	To assess the status of pharmacovigilance structure, processes, outcomes and impact in the South-South zone of Nigeria using the WHO PV indicators	Cross-sectional descriptive study	South-South Zone of Nigeria	Regional health facilities (hospitals)	6 Hospitals	Structured interviews with focal pharmacovigilance persons or committees in hospitals and review of hospital records	Modified WHO Pharmacovigilance Indicators (Core Indicators)	Background information, structural indicators, process indicators, outcome/impact indicators	The absence of trained PV personnel hindered the provision of results for the PV process indicators. Structural PV indicators fail to fully capture the pharmacovigilance system's functionality. Overall poor documentation limited the indicators' derivation. Outcome/impact indicator derivation required an in-depth survey which young PV systems are unable to execute. Need for a scoring system to quantify the indices to highlight deficiencies in numerical terms	33
Qato [49]	To describe the current landscape of pharmacovigilance in the Arab and Eastern Mediterranean (EM) region	Descriptive cross-sectional study	Arab and Eastern Mediterranean Region countries	National Pharmacovigilance Centre	21 countries: Afghanistan (AF), Algeria (AL), Comoros Islands (CO), Djibouti (DJ) (excluded from final mean calculations), Egypt (EG), Jordan (JO), Iran (IR), Iraq (IQ), Kuwait (KW), Libya (LB), Lebanon (LE), Morocco (MA), Oman (OM), Pakistan (PK), Palestine (PA), Qatar (QA), Saudi Arabia (KSA), Sudan (SU), Tunisia (TN), the UAE, Yemen (YE)	Self-administered questionnaires by pharmacovigilance leadership (official national contact for the WHO Programme for International Drug Monitoring (PIDM))	Combination of WHO Pharmacovigilance Indicators and Indicator-Based Pharmacovigilance Assessment Tool (IPAT)	Three domains of pharmacovigilance performance: Structure, process, and impact	Not all countries in the geographical region of interest were represented either due to non-response or incomplete responses to the questionnaire. The survey was only developed in English. Potential for reporting bias	31
Rorig and de Oliveira [57]	To evaluate the implementation and operation of the pharmacovigilance programme in the pharmaceutical industry	Not reported	Brazil	Pharmaceutical companies	50 companies	Self-administered questionnaire by pharmaceutical companies' PV sector, regulatory affairs sector, or customer support service	Not reported	1—Company identification, its origin and the characterisation or absence of a PV programme; 2—Information relating to factors required for PV programme implementation; 3—Pharmacovigilance programme results, and information about notifications reception and how this was treated	Not reported	25
Shin et al. [55]	To survey the collection and management of adverse effect reports in 21 Asia–Pacific Economic Cooperation (APEC) countries, compare the PV status and systems by country, and finally, to harmonise PV regulation in the APEC region	Not reported	Asia‐Pacific Economic Cooperation (APEC) region countries	National Pharmacovigilance Centre	15 countries: Australia (AU), Brunei (BN), Chile (CL), Indonesia (ID), Malaysia (MY), Mexico (MX), Papua New Guinea (PG), Peru (PE), Philippines (PH), Singapore (SG), Taiwan (TW), Thailand (TH), Japan (JP), South Korea (SK), and the USA	Self-administered questionnaires by heads of PV teams from PV agencies	Modified WHO Pharmacovigilance Indicators	Three domains: Structure, process, and outcome of pharmacovigilance system	Not all countries in the region responded to the survey. Did not include all questions and answers from WHO's PV indicators. The tendency for arbitrary interpretation regarding questions on regular pharmacovigilance education	31
Suwankesawong et al. [53]	To explore the current landscape and identify challenges in PV activities among Association of Southeast Asian Nations (ASEAN) countries	Cross-sectional study	ASEAN countries: Brunei Darussalam, Cambodia, Indonesia, Lao People’s Democratic Republic (PDR), Malaysia, Myanmar, Philippines, Singapore, Thailand and Vietnam	National Pharmacovigilance Centre	8 countries: Cambodia (KH), Indonesia (ID), Laos (LA), Malaysia (MY), the Philippines (PH), Singapore (SG), Thailand (TH), and Vietnam (VT)	Self-administered questionnaire by ASEAN countries' PV representatives and contact persons	WHO minimum requirements for a functional national pharmacovigilance system	PV systems' function and performance were measured and compared based on: Indicators related to the average numbers of individual case safety reports (ICSR), presence of signal detection activities and subsequent action, contributions to VigiBase	Application of WHO requirements to national PV systems only, therefore findings may not be generalisable to pharmacovigilance in the entire community	31
Wilbur [50]	To inventory national pharmacovigilance programmes in place for Arabic-speaking countries in the Middle East	Not reported	Arabic-speaking Middle Eastern countries	National Pharmacovigilance Centre	11 countries: Bahrain (BH), Egypt (EG), Iraq (IQ), Jordan (JO), Kingdom of Saudi Arabia (KSA), Kuwait (KW), Oman (OM), Palestine (PA), Qatar (QA), United Arab Emirates (UAE), and Yemen (YE)	Self-administered questionnaire by the head of centres responsible for medication safety	Uppsala Monitoring Centre Assessment of Country Pharmacovigilance Situation questionnaire (February 2008)	General programme information; level of support; PV activities; suspected ADR reporting and subsequent data use; and medication safety advocacy	Certain responses may be different since the original deployment of the questionnaire. The accuracy and completeness of the information provided could be affected depending on the individual completing the questionnaire. Not all countries formally participated so regional situations are not fully described	24
Zhang et al. [56]	To assess the current status of ADR reporting and monitoring in pharmaceutical manufacturers, drugstores, and medical institutions in China	Cross-sectional study	Chinese provinces (East: Jiangsu and Guangdong; West: Shaanxi and Sichuan; and Centre: Henan and Hebei)	Pharmaceutical manufacturers', drugstores', and medical institutions' pharmacovigilance systems	589 institutions (194 pharmaceutical manufacturers, 191 drugstores, and 204 medical institutions)	Self-administered questionnaire by ADR reporters in charge of drug safety (e.g. heads of vigilance units and drug safety coordinators) at Pharmaceutical manufacturers, drugstores, and medical institutions	A questionnaire based on previous studies	1—Current status of the ADR monitoring system; 2—Basic resources for ADR reporting; 3—ADR reporting; and 4- Other PV activities	Data might not fully reflect current adverse drug reaction monitoring and reporting systems in China. It was assumed that the respondents had full access to all current, relevant information. The information supplied by respondents was not verified or validated. The study did not target all the adverse drug reaction reporting and monitoring institutions or all 34 provinces in China. Only three institution types were included, and data collection focussed on the institutional level rather than the individual level. Low response rate	32

Ten studies employed self-completion questionnaires for data collection [45, 48–53, 55–57], and nine employed mixed-methods [37–41, 43, 44, 46, 47] including interviewer-administered questionnaires alongside a documentary review. Two studies [42, 54] employed only qualitative methods including interviews and literature or documentary review. Sixteen studies [37–47, 49, 53–57] evaluated or assessed PV practice or performance. The remaining five studies [48, 50–52, 55] surveyed or provided an overview of countries’ PV situation and offered insights into the maturity of PV systems.

Eight studies [39, 44, 48, 50, 52–55] focussed on national PV centre(s), while three [37, 38, 41] took more of a system-wide approach by also including other levels, i.e. healthcare facilities and PHPs. Three studies [43, 46, 51] focussed on PV at the regional level within a country. Five studies [40, 45, 47, 56, 57] focussed on PV in stakeholder institutions including pharmaceutical companies/manufacturers, Public Health Programmes (PHPs), drugstores and medical institutions.

Thirteen studies [37–44, 46, 47, 49, 53, 55] employed an analytical approach that relied on the use of a framework. The most frequently used frameworks (n = 3) used were the IPAT framework [37, 38, 41] and the WHO PV indicators [46, 47, 55]. Two studies used the East African Community (EAC) harmonised pharmacovigilance indicators tool [39, 40] and two used the WHO minimum requirements for a functional PV system [42, 53]. Two studies [43, 44] employed the Centres for Disease Control and Prevention (CDC) updated guidelines for evaluating public health surveillance systems [58] alongside the WHO PV indicators [30]. One study employed a framework that combined indicators from the IPAT and the WHO PV indicators [49].

### Study Quality

Using Hawker et al.’s [34] nine-item checklist, the overall quality of included studies was deemed as ‘medium’ for seven and ‘high’ for 14. See Online Resource 3 for detailed scoring. The lowest scoring parameter was “ethics and bias” (Average = 1.9, Standard Deviation ± 0.6); the highest scoring parameter was “abstract and title” (3.9 ± 0.3). The methods used were considered appropriate for all included studies; however, seven did not provide sufficient detail on the data collection and recording process [38, 44, 45, 50–52, 57]. Clear sample justification and approaches were only described in three studies [43, 44, 46]. Only three studies [45, 50, 57] were rated poorly or very poorly with respect to data analysis due to limited or no detail. Apart from one study [51], studies provided clear descriptions of findings. Only three studies [41–43] detailed ethical issues such as confidentiality, sensitivity and consent. No studies described or acknowledged researcher bias/reflexivity. Study transferability or generalisability was affected by the use of small sample sizes [37, 41], survey non-response [45, 48–50, 55], focus on the national PV centre [53], the institutional level rather than the individual (Healthcare Professional (HCP) or patient) level, exclusion of some types of institutions [56] and non-testing of questionnaire reliability [52]. Only four studies [41, 52–54] achieved a score of 4 for the “implications and usefulness” parameter by making suggestions for future research and implications for policy and/or practice.

The main limitation described by the reviewed studies related to information validity and completeness. Eight studies [39, 40, 42, 43, 48, 50, 52, 56] cited limitations that included pertinent data missing, reliance on the accuracy of information provided or inability to verify or validate information. The second limitation was related to the collected data's currency [39, 48, 50, 56].

Finally, two studies [41, 46] reported limitations related to the evaluation tools used to evaluate PV performance. Kabore et al. [41] highlighted four limitations inherent to the IPAT including 1—Its sensitivity and specificity had not been established, 2—Possible imprecision in the quantification of responses in the scoring process, 3—The assessment’s reliance on respondents’ declarations and 4—The necessity of local adaptation due to the tool's limited testing and validation. Two studies [46, 47] raised limitations of using the WHO PV indicators including lack of trained personnel, poor documentation and the need for in-depth surveys which nascent systems are unable to execute. Furthermore, the WHO PV indicators were said to lack a scoring system that could quantify the indices thereby highlighting system deficiencies numerically [46].

### Studies’ Coverage of WHO Pharmacovigilance Indicators

**Fig. 2:**
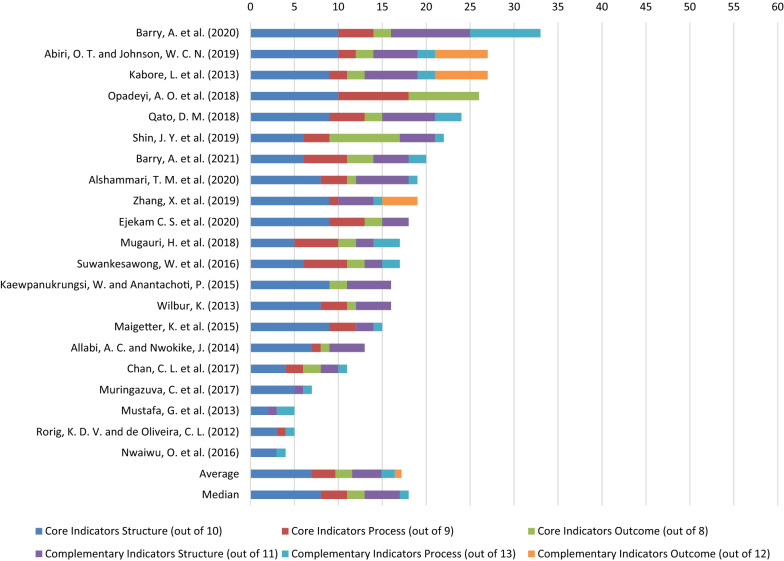
Included studies’ aggregate scores (out of 63) for coverage of WHO pharmacovigilance indicators

When investigating the number of all 63 WHO PV indicators, the studies achieved an average score of 17.2 (see Fig. 2). The highest score was 33.0 [39] and the lowest was 4.0 [45]. Studies placed a higher emphasis on evaluating ‘Core’ compared to ‘Complementary’ indicators as demonstrated by the median and average scores obtained for ‘Core’ (12.0 and 11.6/27, respectively) versus 4.0 and 5.6/36 for ‘Complementary’. Studies obtained higher median and average scores for ‘Structural’ indicators (8.0 and 7.0/10 for ‘Core’ and 4.0 and 3.3/11 for ‘Complementary’, respectively) compared to ‘Process’ (3.0 and 2.7/9 for ‘Core’ along with 1.0 and 1.5/13 for ‘Complementary’, respectively) and ‘Outcome’ indicators (2.0 and 1.9/8 for ‘Core’ and 0 and 0.8/12 for ‘Complementary’). Further detail is supplied in Online Resource 4.

### Regions’ and Countries’ Pharmacovigilance Performance

#### Total Pharmacovigilance System Performance

**Fig. 3:**
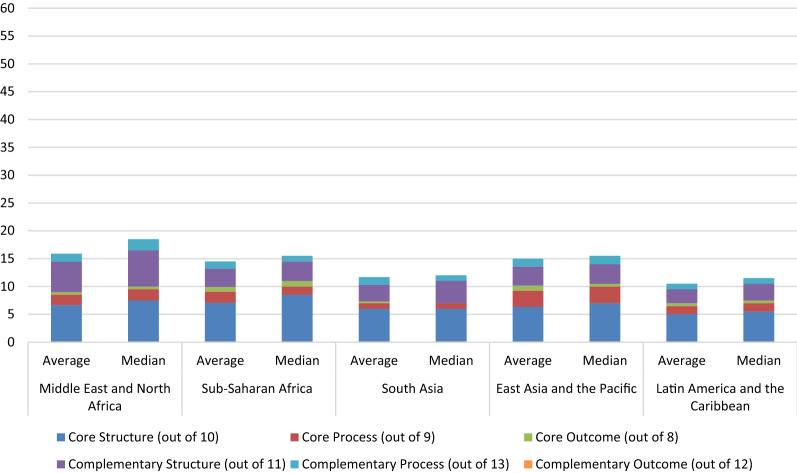
Aggregate scores (out of 63) of studied countries’ pharmacovigilance systems by region

**Fig. 4:**
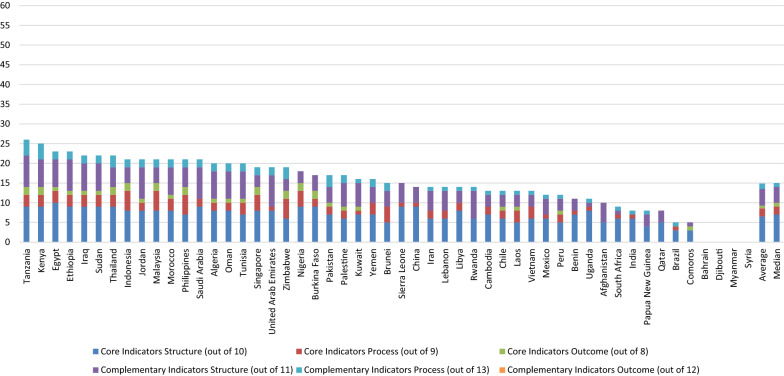
Aggregate scores (out of 63) of studied countries’ pharmacovigilance systems

The average and median scores achieved by all countries were 14.86 and 15.0/63, respectively. Although 51% of countries had a higher-than-average total score and 49% had a score above the median, none of them achieved more than 40% of the WHO indicators. The Middle East and North Africa achieved the highest average total score (15.89), and Latin America and the Caribbean the lowest (10.5). In comparison, the highest median score was achieved by the Middle East and North Africa (18.0), and the lowest was achieved by South Asia (10.0). The highest achieving country was Tanzania (26.0). Bahrain, Syria, Djibouti and Myanmar all scored zero. See Figs. 3 and 4 for the regions’ and countries’ aggregate scores, respectively, Online Resource 4 for detailed information relating to each indicator, and Online Resource 5 for detailed information on aggregate scores.

#### Core Indicators Performance

Out of a possible score of 27 for ‘Core’ indicators, the average was 9.27 while the median was 9.0. East Asia and the Pacific achieved the highest average score (10.17), whereas South Asia had the lowest (7.3). On the other hand, in terms of the median score, the highest was observed in Sub-Saharan Africa (11.5). And the lowest was in South Asia (7.0). The highest scoring countries among the different regions were Nigeria, Indonesia and Malaysia (15.0), whereas Bahrain, Syria, Djibouti and Myanmar scored zero.

##### Structural Indicators

For ‘Core Structural’ indicators, the average score for the 51 countries was 6.5 and the median was 7.0. The highest average and median scores, regionally, were observed in Sub-Saharan Africa (7.07 and 8.5, respectively), whereas the lowest were observed in Latin America and the Caribbean (5.0 and 5.5, respectively). Egypt had the highest country-level score (10.0) while Bahrain and Syria, Djibouti and Myanmar scored zero.

A facility for carrying out PV activities was reported as existing in 92% of countries, and PV regulations existed in 80% of countries. There were inconsistencies in the reported information concerning PV regulations in Oman, Yemen and Cambodia. In Oman, two studies [48, 50] reported that such regulations were present, whereas a third [49] reported they were absent. In Yemen, Qato [49] reported the presence of regulations, whereas Alshammari et al. [48] indicated the opposite. For Cambodia, conflicting information was reported by Suwankesawong et al. [53] and Chan et al. [52]. In all such cases, the latest published results were adopted.

Concerning resources, regular financial provision for conducting PV activities was reported as present in only 35% of countries, most of which were among the highest achieving countries overall. There was an inconsistency in the information provided for this indicator in Oman and the United Arab Emirates (UAE) with two studies [48, 50] stating that this was present, and one [49] that it was not. In terms of human resources, 75% of countries were found to possess dedicated staff carrying out PV activities.

Most countries (86%) were found to possess a standardised ADR reporting form. However, it was only highlighted in 16 countries whether the form included medication errors; counterfeit/substandard medicines; therapeutic ineffectiveness; misuse, abuse, or dependence on medicines; or reporting by the general public.

For only four countries (China, Egypt, Ethiopia and Uganda) was it reported that PV was incorporated into the national HCP curriculum. In 22 countries (43%), it was either unknown if a PV information dissemination mechanism existed, or it did not exist. Sixty-three per cent of countries had a PV advisory committee. Information regarding this indicator was inconsistent between Qato [49] and Alshammari et al. [48] with the former reporting Jordan and Tunisia possessed an advisory committee, the latter reporting the opposite.

##### Process Indicators

The overall average and median scores for ‘Core Process’ indicators were 2.06 and 2.0/9, respectively. The highest average score was in East Asia and the Pacific (2.9), whereas South Asia (1.0) achieved the lowest. Similarly, in terms of the median score, East Asia and the Pacific (3.0) was the highest while South Asia (1.0) was the lowest. No country achieved a higher score than Malaysia (7.0), while seven countries scored zero.

The absolute number of ADR reports received per year by the countries’ PV system ranged from zero (Afghanistan, Bahrain, Comoros, Qatar, and Rwanda) to 50,000 (Thailand). Most countries (*n* = 27) received less than 10,000 reports per year, with Iran reporting the highest yearly rate (7532 reports) and Laos and Lebanon reporting the lowest (3 reports). Only four countries reported receiving 10,000 reports or more yearly, namely China (32,513 reports), Malaysia (10,000 reports), Singapore (21,000 reports) and Thailand (50,000 reports). The remaining 20 countries either did not receive any reports or no data were provided.

The number of ADR reports increased over time in 12 countries (Algeria, Cambodia, Egypt, Iraq, Jordan, Kuwait, Morocco, Oman, Palestine, Saudi Arabia, Tunisia and Yemen), whereas they decreased in eight countries (Laos, Malaysia, Philippines, Singapore, Sudan, Thailand, the UAE and Vietnam). The percentage of total annual reports satisfactorily completed and submitted to the PV centre was reported only in Nigeria (maximum of 84.6%).

Only Singapore and Thailand reported cumulative numbers of reports as more than 100,000, while 17 countries had fewer than 20,000 reports cumulatively. Some inconsistencies for this indicator were reported by Suwankesawong et al. [53] and Chan et al. [52] for Malaysia, the Philippines, Singapore and Vietnam, with the numbers reported by the former higher than the latter.

Overall, the provision of ADR reporting feedback was poor, with all the countries either not performing this or no information being provided. Documentation of causality assessment was also poor, with only Ethiopia (2%), Kenya (5.5%), Tanzania (97%) and Zimbabwe (100%) reportedly performing this. The percentage of reports submitted to WHO was reported only in Vietnam (28%) and Zimbabwe (86%).

Among the countries which reported performing active surveillance, Algeria was the most active with 100 projects followed by Tunisia and Morocco with 50 and 10 activities, respectively. All remaining countries had fewer than seven.

##### Outcome Indicators

The average and mean scores overall for the ‘Core Outcome’ indicators were 0.69 and 1.0/8, respectively. Countries from East Asia and the Pacific (0.92) had the highest average score collectively, whereas South Asia (0.33) had the lowest. In terms of the median score, sub-Saharan Africa (1.0) had the highest, whereas South Asia (zero) had the lowest. Nine countries achieved the highest score (2.0), while 25 countries only scored zero.

Signal detection was reported to have occurred in 10 countries, with the highest number observed in Kenya (31 signals), whereas seven countries scored zero. The reported number of signals detected was above 10 in only three countries: Kenya, Tanzania (25 signals) and Singapore (20 signals). Among the 23 countries where information regarding the number of regulatory actions taken was reported, the highest number of actions taken was in Egypt (930 actions), whereas in 15 countries, no actions had been taken.

The number of medicine-related hospital admissions per 1000 admissions was only reported in Nigeria and ranged from 0.01 to 1.7. The reporting of pertinent data regarding the remaining five Core Outcome indicators (CO3–CO8) was inadequate as no information was provided for any of the countries.

#### Complementary Indicators Performance

For ‘Complementary’ indicators, the overall average and median scores were 5.59 and 6.0/36, respectively. The Middle East and North Africa (6.89 and 8.5, respectively) achieved the highest average and median scores among the regions, whereas Latin America and the Caribbean (3.5 and 4.0, respectively) achieved the lowest. The highest scoring country was Tanzania (12.0), whereas Bahrain, Syria, Djibouti and Myanmar scored zero.

##### Structural Indicators

For ‘Complementary Structural’ indicators, the average and mean scores were 4.24 and 4.0/11, respectively. The highest average and median scores were achieved by the Middle East and North Africa (5.44 and 6.0, respectively), whereas Latin America and the Caribbean (2.5 and 3.0, respectively) had the lowest. Five countries achieved a score of 8.0, namely Jordan, Saudi Arabia, the UAE, Ethiopia and Tanzania. Seven countries scored zero.

Three-fourths of the countries were reported to possess dedicated computer facilities to carry out PV activities as well as a database for storing and managing PV information. There was inconsistency in the data reported for Libya, with Qato [49] indicating the presence of a computer, whereas Alshammari et al. [48] reported it absent. It was indicated that in 47% of the countries, functioning communication facilities such as telephone, fax, or internet were available. A library containing reference materials on drug safety was found to be available in only 19 countries. For all the countries, it was either reported that they did not have a source of data on consumption and prescription of medicines, or no information was available.

In all 51 countries investigated, it was either reported that web-based PV training tools for both HCPs and the public were not available, or no information was reported. It was found that in 30 (60%) of countries training courses for HCPs were organised by the PV centre. There was insufficient information about the availability of training courses for the public in all countries. Less than half (41% and 49%, respectively) of countries possessed a programme with a laboratory for monitoring drug quality or mandated MAHs to submit Periodic Safety Update Reports (PSURs). Only 8% of countries had an essential medicines list and only 18% used PV data in developing treatment guidelines.

##### Process Indicators

The 51 countries achieved average and median scores of 1.4 and 1.0/13, respectively, for the ‘Complementary Process’ indicators. Regionally, the highest average and median scores were achieved by the Middle East and North Africa (1.44 and 2.0, respectively), while the lowest scores were achieved by Latin America and the Caribbean (both 1.0). The highest total scores were achieved by Kenya and Tanzania (both 4.0), while 12 countries scored zero.

Data regarding the percentage of healthcare facilities possessing a functional PV unit (i.e. submitting ≥ 10 reports annually to the PV centre) was reported for seven countries. However, only three of these reported a number above zero (Kenya 0.14%, Tanzania 0.26% and Zimbabwe 2.2%).

In terms of the total number of reports received per million population; it was found that Singapore had the highest number (3853 reports/year/million population), while Laos had the lowest (0.4 reports/year/million population). In 17 countries, it was indicated that HCPs represented the primary source of submitted ADR reports. Medical doctors were reported as the primary HCPs to submit ADR reports in five countries, namely Lebanon (100%), Libya (50%), Morocco (50%), Tunisia (96%) and Yemen (90%). In eight countries, manufacturers were found to be the primary source of ADR reports, namely Algeria (71%), Jordan (90%), Kuwait (93%), Mexico (59%), Pakistan 88%), Palestine (100%), Saudi Arabia (50%) and the UAE (72%).

The number of HCPs who received face-to-face training over the previous year was only reported in Ethiopia (90,814), Tanzania (76,405), Rwanda (43,725) and Kenya (8706).

No information was found in any of the studies concerning the ‘Complementary Process’ indicators 4, 6 and 9–13.

##### Outcome Indicators

Out of a possible score of 12, the overall average and median scores achieved for the ‘Complementary Outcome’ indicators of the studied countries were both zero, with no information reported concerning these indicators.

## Discussion

To the best of the authors’ knowledge, this is the first systematic review of studies focussing on PV system performance in developing countries. The review included 21 studies covering 51 countries from different regions across the globe. Using the WHO PV indicators (both ‘Core’ and ‘Complementary’) [30] as a framework, this review focussed on identifying the areas of strength and weakness within these countries’ PV systems. The review also helped identify where different developing countries’ systems lay on the performance level spectrum. Moreover, the features associated with better performing systems were highlighted. The insights from this review can be used to inform recommendations for addressing areas requiring intervention or modification, particularly within countries with PV systems at a nascent stage of development.

The review revealed a lack of standardisation regarding the methods of evaluating PV systems. While some studies focussed on the WHO indicators, others used assessment tools developed by other organisations including the United States Agency for International Development (USAID), East African Community (EAC), the United States Centre for Disease Control (CDC) or some combination of these. The review also found that, overall, both studies’ coverage of the WHO PV indicators and developing countries’ PV system performance were both low. Furthermore, there was a mix of some indicators which were present in most or all studies/countries, while others were universally absent or only sporadically present. Generally, indicators that were either universally absent or only sporadically present in the studies/countries in this review belonged to the ‘Process’ and ‘Outcome’ indicator classes. In terms of the reviewed studies, both the ‘Complementary Process’ and ‘Complementary Outcome’ indicators’ presence was mixed with some being universally absent (e.g. number of reports from each registered pharmaceutical company received by the NPVC in the previous year and cost savings attributed to PV activities, respectively) and others being sporadically present (e.g. number of face-to-face training sessions in PV organised in the previous year and average number of medicines per prescription, respectively). Most of the ‘Core Process’ and ‘Core Outcome’ and ‘Complementary Structural’ indicators were sporadically present (e.g. percentage of reports on medication errors reported in the previous year, average cost of treatment of medicine-related illness and existence of an essential medicines list which is in use, respectively), whereas most of the ‘Core Structural’ indicators were frequently present (e.g. the NPVC has human resources to carry out its functions properly) and only a few were sporadically present (incorporation of PV into the national curriculum of the various HCPs).

In terms of the studied countries, all the ‘Complementary Outcome’ (e.g. percentage of medicines in the pharmaceutical market that is counterfeit/substandard) indicators were universally absent. The ‘Core Outcome’ and ‘Complementary Process’ indicators' presence was found to be mixed with some being universally absent (e.g. number of medicine-related deaths and percentage of MAHs submitting PSURs to the NMRA, respectively) while others were sporadically present (e.g. number of signals detected in the past five years and percentage of HCPs aware of and knowledgeable about ADRs per facility). Most of the ‘Core process’ (e.g. percentage of submitted ADR reports acknowledgement or issued feedback) indicators were found to be sporadically present. Therefore, PV system performance was found to be low in terms of the ‘Process’ and ‘Outcome’ indicators. This reflects immaturity and the inability to collect and utilise local data to identify signals of drug-related problems and to support regulatory decisions [22, 59–61].

With regard to ‘Structural’ indicators, most of the ‘Core’ (e.g. an organised centre to oversee PV activities) and some of the ‘Complementary’ (e.g. existence of a dedicated computer for PV activities) structural indicators were found to be frequently present among the studied countries. Hence, performance with respect to the class of ‘Structural’ indicators was relatively high. This points to government policymakers taking active steps towards establishing a PV system as a means of improving drug safety [3, 21].

High-performing PV systems in developing countries in this review were distinguished by the presence of a budget specifically earmarked for PV, a means of communicating drug safety information to stakeholders (e.g. a newsletter or website) and technical assistance via an advisory committee. On the other hand, lack of incorporation of PV into the national curriculum of HCPs and underreporting of ADRs plagued both high- and low-performing systems. This suggests that strengthening PV systems in developing countries requires targeted measures addressing these factors. In what follows, this review’s key findings described above will be discussed in more detail in the context of the WHO PV indicators[30] and existing research.

The 63 indicators developed by the WHO were not all assessed in the included studies. This meant that the data collection process in some instances necessitated extracting data from other sections of the studies such as the ‘Background’ or ‘Discussion’. In other instances, inferences were made for certain indicators based on information provided for others. A notable example was inferring the presence of a computer for PV activities when it was indicated that a computerised case report management system existed. Evaluation is defined as the systematic and objective assessment of the relevance, adequacy, progress, efficiency, effectiveness and impact of a course of action in relation to objectives while considering the resources and facilities that have been deployed [62]. An evaluation based only on a few indicators is not likely to provide a complete, unbiased evaluation of the system since multiple indicators are needed for tracking the system’s implementation and effects [58]. While the optimal number of indicators required to perform a proper assessment is likely to vary depending on the evaluation’s objectives, it could be argued that, based on definition, addressing the full set of ‘Core’ indicators should be required to provide a satisfactory evaluation [33].

This review found that the presence of a dedicated budget for PV was associated with higher system performance [30, 59, 60, 63]. The absence of sustained funding for PV hinders effective system operation since it prevents the development of the necessary infrastructure [64]. According to the WHO, funding is what allows the carrying out of PV activities in the setting [30] and it “signifies a gesture, the commitment and political will of the sponsors and the general importance given to PV” (p. 20) [30]. It is only when the other structural components of a PV system are paired with a regular and sustainable budget that real action and long-term planning can be achieved [65–67]. Any investment in PV should consider the substantial diversity in country characteristics such as size and population as well as the anticipated rate at which the system is going to generate reports [21, 68].

In this review, countries that had a PV information dissemination tool as part of the system achieved higher-performance scores than those that did not. The WHO indicates that an expected function of a country’s PV system is the effective dissemination of information related to medicines’ safety to both HCPs and the public [3, 30, 69]. The lack of such a tool in many developing countries systems points to the absence of clear routine and crises communication strategies [30]. The use of a drug bulletin has been cited as an effective tool for improving safety communication as well as increasing ADR reporting [70–72].

A feature of better performing PV systems was the presence of a PV (or ADR) advisory committee. The WHO views the existence of such a committee as essential given its influential role in developing a clear communication strategy as well as providing technical assistance to the drug regulatory process. The absence of such a committee negatively impacts system processes such as causality assessment, risk assessment and management, as well as outcomes such as communication of recommendations on safety issues and regulatory actions. Evidence from developed countries has demonstrated the value of such a committee’s scientific and clinical advice to support and promote drug safety [73, 74].

PV was found to be absent from the national curricula of HCPs in most of the countries studied, which may explain low levels of competency regarding PV and ADR reporting [75]. Studies have demonstrated that the implementation of PV-related training as a module or course for HCP students has a positive effect on their PV knowledge [76–78] and sensitises HCPs to issues regarding drug safety [30].

This review found that ADR reporting rates were low overall, suggesting underreporting by ADR reporters [23, 79], which may be partly due to the passive nature of the reporting systems in these [59]. Underreporting points to the PV system’s inability to collate data on the safety, quality and effectiveness of marketed drugs that have not been tested outside the confines of clinical trials. Consequently, system processes and outcomes, including data analysis, signal identification, regulatory actions, and communication and feedback mechanisms, will remain stagnant. The WHO’s guidance points to the number of ADR reports received by the system as being an indicator of PV activity in the setting, the awareness of ADRs and the willingness of HCPs to report [30]. Despite underreporting being a significant barrier to the effective functioning of PV systems in both developing and developed countries [65, 74], reporting rates have been found to be lower in developing countries than in developed ones [80]. Based on international evidence, it is reasonable to expect a developed system to target an annual reporting rate of 300 reports per million inhabitants [81]. Countries struggling with underreporting should utilise the WHO’s global database (VigiBase) as a reference for monitoring drug-related problems [60]. Furthermore, data from countries with similar population characteristics and co-morbidities receiving smaller numbers of ADR can be gathered into a single database which would allow an analysis of the pooled data to provide relevant solutions [60, 64].

This review has a few limitations. First, the included studies were very heterogeneous and differed in their aim, structure, content, method of evaluation and targeted level of PV system/activity, which may limit the extent of the findings’ generalisability. This was partially overcome by applying the WHO indicators as a means of standardising the extracted information. Second, a limitation of the WHO PV indicators is the lack of a scoring system to quantifiably measure PV system performance. This was overcome by the development of a scoring system thus enabling a comparison of a country’s PV system performance status against the WHO PV indicators and that of other countries.

## Conclusion

This is the first systematic review that focuses on studies that evaluate PV performance and activities in developing countries, using WHO PV indicators. The included studies provide an in-depth understanding of the various factors affecting PV system performance and activities. This study’s findings demonstrate that a multistakeholder approach towards strengthening PV systems in developing countries is required and the necessity of resource and data consolidation and the establishment of regional collaborations to assist PV systems that are in their nascent stage. Furthermore, it highlights the need for applying a holistic approach that takes into account the resources and infrastructure available when addressing the policy and programmatic gaps in each country.

## Supplementary Information

Below is the link to the electronic supplementary material.

Supplementary file1 (PDF 138 kb)

Supplementary file2 (PDF 168 kb)

Supplementary file3 (XLSX 32 kb)

Supplementary file4 (XLSX 101 kb)

Supplementary file5 (XLSX 93 kb)
