# Supplementary material for: A Systematic Review of Pharmacovigilance Systems in Developing Countries Using the WHO Pharmacovigilance Indicators
Source: Ther Innov Regul Sci. 2022 Jun 3;56(5):717–43. doi: 10.1007/s43441-022-00415-y (PMC9356965; doi:10.1007/s43441-022-00415-y)
Supplement: Supplementary file 2 — Supplementary file2 (PDF 168 kb) [file 43441_2022_415_MOESM2_ESM.pdf]

A systematic review of pharmacovigilance systems in developing countries using the WHO pharmacovigilance indicators

Hamza Y. Garashi, Douglas T. Steinke, Ellen I. Schafheutle

Correspondence: hamza.garashi@postgrad.manchester.ac.uk

Division of Pharmacy and Optometry, School of Health Sciences, Faculty of Biology, Medicine and Health, The University of Manchester, Manchester M13 9PT, UK

Pharmacovigilance System Implementation Evaluation Search Strategy

Ovid MEDLINE(R) <1946 to July Week 2 2021>

#	Search Term	Number of Results
1	Pharmacovigilance/	2435
2	Adverse Drug Reaction Reporting Systems/	7982
3	Product Surveillance, Postmarketing/	7355
4	Evaluation Studies as Topic/	122298
5	Program Evaluation/	65198
6	Benchmarking/	14389
7	Outcome Assessment, Health Care/	76695
8	Process Assessment, Health Care/	4879
9	"Outcome and Process Assessment, Health Care"/	28091
10	monitor*.mp.	907041
11	1 or 2 or 3	15886
12	4 or 5 or 6 or 7 or 8 or 9 or 10	1193601
13	11 and 12	3265
14	limit 14 to (english language and yr="2012 -Current")	1231

Embase <1974 to 2021 July 16>

#	Search Term	Number of Results
1	Pharmacovigilance/	2457
2	drug surveillance program/	26286
3	postmarketing surveillance/	11672
4	evaluation study/	49798
5	program evaluation/	16604
6	evaluation research/	2057
7	benchmarking/	6728
8	monitoring/	169687
9	outcome assessment/	600140
10	1 or 2 or 3	39215
11	4 or 5 or 6 or 7 or 8 or 9	838370
12	10 and 11	3166
13	limit 12 to (english language and yr="2012 -Current")	2407

Web of Science

Search Terms	Results
ALL=(pharmacovigilance OR "Postmarketing surveillance" OR "Drug surveillance program" OR "adverse drug reaction reporting systems")	21,692
ALL=(Evaluat* OR Monitor* OR Assess* OR Benchmark*)	11,055,069
ALL=(pharmacovigilance OR "Postmarketing surveillance" OR "Drug surveillance program" OR "adverse drug reaction reporting systems") AND ALL=(Evaluat* OR Monitor* OR Assess* OR Benchmark*)	8,595
(ALL=(pharmacovigilance OR "Postmarketing surveillance" OR "Drug surveillance program" OR "adverse drug reaction reporting systems") AND ALL=(Evaluat* OR Monitor* OR Assess* OR Benchmark*)) AND (PY==("2012" OR "2013" OR "2014" OR "2015" OR "2016" OR "2017" OR "2018" OR "2019" OR "2020" OR "2021"))	5,918
(ALL=(pharmacovigilance OR "Postmarketing surveillance" OR "Drug surveillance program" OR "adverse drug reaction reporting systems") AND ALL=(Evaluat* OR Monitor* OR Assess* OR Benchmark*)) AND (PY==("2012" OR "2013" OR "2014" OR "2015" OR "2016" OR "2017" OR "2018" OR "2019" OR "2020" OR "2021") AND LA==("ENGLISH"))	5,663

CINAHL Plus

Search ID#	Search Terms	Actions
S14	S10 AND S11<br>**Limiters** - Publication Year: 2012-2021<br>**Narrow by Language:** - english	1,441
S13	S10 AND S11<br>**Narrow by Language:** - english	2,394
S12	S10 AND S11	2,427
S11	S4 OR S5 OR S6 OR S7 OR S8 OR S9	564,727
S10	S1 OR S2 OR S3	14,642
S9	"monitor*"	193,652
S8	(MH "Process Assessment (Health Care)")	4,816
S7	(MH "Outcome Assessment")	46,123
S6	(MH "Benchmarking")	7,620
S5	(MH "Evaluation Research")	300,110
S4	(MH "Program Evaluation")	43,963
S3	"adverse drug reaction reporting systems"	7,053
S2	(MH "Drug Evaluation")	6,907
S1	(MH "Pharmacovigilance")	1,356
